# Parkinson's disease and obstructive sleep apnea

**DOI:** 10.17179/excli2021-3359

**Published:** 2021-02-23

**Authors:** Tomoyuki Kawada

**Affiliations:** 1 Department of Hygiene and Public Health, Nippon Medical School, 1-1-5 Sendagi, Bunkyo-Ku, Tokyo 113-8602, Japan

## ⁯

***Dear Editor,***

Sleep disorders are common in Parkinson's disease (PD), and PD reduces sleep quality and quality of life in patients and their caregivers (Zuzuárregui and During, 2020[6]). I present information regarding the association with special reference to obstructive sleep apnea (OSA).

First, Sun et al. (2020[4]) conducted a meta-analysis, and the pooled hazard ratio (95 % confidence interval [CI]) of patients with OSA for the occurrence of PD was 1.59 (1.36-1.85). In contrast, the incidence of OSA did not increase in patients with PD, presenting a pooled odds ratio (95 % CI) of 0.89 (0.53-1.49). Zeng et al. (2013[5]) conducted a meta-analysis, and the pooled odds ratio (95 % CI) of PD for OSA was 0.60 (0.44-0.81). These data mean that the protective effect of PD on OSA is inconsistent, and the causal directions should be interpreted with caution.

Second, Shen et al. (2020[3]) clarified that treatment of higher levodopa equivalent dose were protective factors for OSA in patients with PD, presenting intervention effect of PD treatment on OSA. Although OSA risk mechanisms in patients with PD were reviewed from neurodegenerative progression (Kaminska et al., 2015[2]), the associations between PD and OSA should be specified by appropriate adjustments of confounders and current information on the treatment of PD (Büchele et al., 2018[1]).

## Conflict of interest

The author declares no conflict of interest.
